# TNF‐α can promote membrane invasion by activating the MAPK/MMP9 signaling pathway through autocrine in bone‐invasive pituitary adenoma

**DOI:** 10.1111/cns.14749

**Published:** 2024-05-13

**Authors:** Xinzhi Wu, Lei Gong, Bin Li, Jiwei Bai, Chuzhong Li, Yazhuo Zhang, Haibo Zhu

**Affiliations:** ^1^ Beijing Neurosurgical Institute Capital Medical University Beijing China; ^2^ Department of Neurosurgery, Beijing Tiantan Hospital Capital Medical University Beijing China; ^3^ Department of Neurosurgery Peking University People's Hospital Beijing China

**Keywords:** bone invasion, membrane invasion, pituitary adenoma, SPD304, TNF‐α

## Abstract

**Aims:**

A bone‐invasive pituitary adenoma exhibits aggressive behavior, leading to a worse prognosis. We have found that TNF‐α promotes bone invasion by facilitating the differentiation of osteoclasts, however, before bone‐invasive pituitary adenoma invades bone tissue, it needs to penetrate the dura mater, and this mechanism is not yet clear.

**Methods:**

We performed transcriptome microarrays on specimens of bone‐invasive pituitary adenomas (BIPAs) and noninvasive pituitary adenomas (NIPAs) and conducted differential expressed gene analysis and enrichment analysis. We altered the expression of TNF‐α through plasmids, then validated the effects of TNF‐α on GH3 cells and verified the efficacy of the TNF‐α inhibitor SPD304. Finally, the effects of TNF‐α were validated in in vivo experiments.

**Results:**

Pathway act work showed that the MAPK pathway was significantly implicated in the pathway network. The expression of TNF‐α, MMP9, and p‐p38 is higher in BIPAs than in NIPAs. Overexpression of TNF‐α elevated the expression of MAPK pathway proteins and MMP9 in GH3 cells, as well as promoted proliferation, migration, and invasion of GH3 cells. Flow cytometry indicated that TNF‐α overexpression increased the G2 phase ratio in GH3 cells and inhibited apoptosis. The expression of MMP9 was reduced after blocking the P38 MAPK pathway; overexpression of MMP9 promoted invasion of GH3 cells. In vivo experiments confirm that the TNF‐α overexpression group has larger tumor volumes. SPD304 was able to suppress the effects caused by TNF‐α overexpression.

**Conclusion:**

Bone‐invasive pituitary adenoma secretes higher levels of TNF‐α, which then acts on itself in an autocrine manner, activating the MAPK pathway and promoting the expression of MMP9, thereby accelerating the membrane invasion process. SPD304 significantly inhibits the effect of TNF‐α and may be applied in the clinical treatment of bone‐invasive pituitary adenoma.

## Graphical abstract

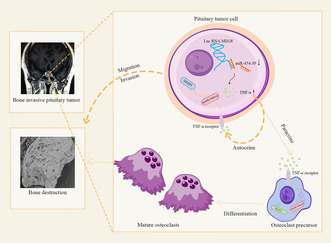

## 1 INTRODUCTION

Pituitary adenomas are benign adenomas originating from the anterior lobe of the pituitary gland, constituting approximately 8%–15% of intracranial adenomas. Despite their benign nature, certain phenotypes demonstrating invasive behavior, particularly bone invasion, exhibit distinct malignant biological characteristics. Consequently, the clinical management of such pituitary adenomas poses numerous challenges.^1^, ^2^ The anatomical structures surrounding the pituitary are complex and consist of vital components, including cranial nerves, the hypothalamus, internal carotid arteries, and the brainstem. Invasive pituitary adenomas can involve the cavernous sinus, sphenoid sinus, suprasellar region, and even structures like the cranial base dura mater and bone (clivus, etc.). This particular trait renders the complete resection of invasive pituitary adenomas challenging, often requiring staged surgeries, adjuvant radiotherapy, or pharmacological interventions.

Research on invasive pituitary adenomas has traditionally focused on involvement of the cavernous sinus and the suprasellar region.^3^, ^4^ However, the clinical characteristics and molecular mechanisms of bone‐invasive pituitary adenoma (BIPA) have not been reported. Following bone invasion, normal bone transforms into numerous bone fragments enveloped by adenoma tissue. Inadequate intraoperative management can directly lead to damage to critical vessels, cranial nerves, the dura mater, or brain tissue, significantly increasing surgical difficulty and risk.

Our preliminary investigation identified TNFα as a pivotal factor in BIPA.^5^ Elevated levels of the long noncoding RNA MEG8 were detected in BIPA, downregulating miR454‐3p and subsequently increasing TNFα expression.^6^ This molecular cascade influences preosteoclasts, promoting their differentiation into osteoclasts, and facilitating bone invasion. Meanwhile, intraoperative visualization revealed localized disruption of the dura mater at the base of the saddle in BIPAs. The tumor protruded from the defective dura mater. This implies that prior to invading bone, tumor cells need to breach the dura mater. The specific mechanisms underlying the invasion of the dura mater remain unclear. We hypothesize that TNF‐α secreted by BIPA cells acts on itself in an autocrine manner, activating the MAPK/MMP9 signaling pathway, promoting the membranous invasion process of BIPA.

In this study, we investigated the roles of TNFα and the MAPK/MMP9 signaling pathway in the membranous invasion process of BIPA. More importantly, we validated a drug that can inhibit the biological effects of TNFα in promoting the proliferation and invasion of pituitary adenomas, providing a novel direction for the treatment of BIPA.

## 2 MATERIALS AND METHODS

### 2.1 Clinical materials and tumor specimens

The criteria for bone invasion included severe destruction of the clivus or anterior skull base on CT and bone destruction observed during the operation or pathological reports showing bone tissue infiltration. BIPAs (*n* = 5) and non‐invasive pituitary adenomas (NIPAs) (*n* = 4) surgical specimens were obtained for transcriptome microarrays. Detailed information about the patients is in Table S1. A total of 40 samples were obtained for the tissue PCR and Western Blot, including 20 BIPAs and 20 NIPAs. All patients involved in this study underwent surgical treatment at Beijing Tiantan Hospital between 2020 and 2022. Within 30 min after resection, tumor specimens were promptly stored in liquid nitrogen for subsequent RNA extraction. This study received approval from the ethics committee of Beijing Tiantan Hospital. All enrolled subjects provided informed consent, and the study was conducted in strict adherence to the principles outlined in the Declaration of Helsinki.

### 2.2 Hematoxylin and eosin staining

The tumor specimens underwent fixation in 10% paraformaldehyde, decalcification in formic acid, and embedding in paraffin. All samples were sectioned at a thickness of 5 mm. The pituitary adenoma specimens were subsequently stained with hematoxylin and eosin (HE).

### 2.3 Differentially expressed genes (DEGs) and functional enrichment analysis

Differentially expressed genes (DEGs) were identified through comparative analysis between the BIPA group and the NIPA group using the R program (version 3.5.3). Further investigation focused on genes exhibiting |fold change| > 2 and a *p*‐value <0.05. DEGs underwent enrichment analysis utilizing both Gene Ontology (GO) and the Kyoto Encyclopedia of Genes and Genomes (KEGG) Pathway Enrichment to predict their biological functions. The pathway interaction analysis was conducted using the ClueGo Cytoscape plugin (version 3.3.0). A GO and KEGG enrichment with an adjusted *p*‐value <0.05 was considered significant.

### 2.4 Cell lines and cell culture

The GH3 rat pituitary cell line was obtained from the American Type Culture Collection (CCL‐82.1, Manassas, VA, USA) and cultured using Ham's F12K medium supplemented with 10% fetal bovine serum (FBS) (Gibco, Waltham, MA, USA). Cells were cultured in a humidified incubator containing 5% CO_2_ at 37°C.

### 2.5 Cell treatment and transfection

The overexpressed plasmids and small interfering RNA (siRNA) for rat TNF‐α and MMP9 were synthesized by GENECHEM (Shanghai, China). Transfections, involving both knockdown and overexpression plasmids, were conducted using Lipofectamine® 3000 Transfection Reagent (Invitrogen, Carlsbad, CA, USA) in accordance with the manufacturer's protocols. After transfection for 24 h, the respective groups were treated with SPD304 (HY‐111255, MedChemExpress) at concentrations of 5 and 8 μM. A P38 MAPK inhibitor (Adezmapimod, MCE, SB203580) was added to GH3 cells 24 h after transfection. Following transfection, cells were incubated for 48 h and subsequently harvested for quantitative (q) PCR and western blot analyses.

### 2.6 Transwell migration and invasion assay

Cell migration and invasion assessments were conducted using a transwell chamber with 8‐μm pore membranes in 24‐well plates (Corning, USA) and invasion chamber with 8‐μm pore membranes in 24‐well plates (Corning, USA). For each assay, 200 μL of cell suspension were placed in the upper chamber of the transwells, creating a density of 20 x 10^4^ cells/well. In the lower chamber, 500 μL of culture medium with 10% FBS were added as a chemoattractant. Cells were then incubated at 37°C and 5% CO2 for 72 h. Post incubation, cells on the membrane's lower surface were stained with crystal violet for 10 min, followed by two PBS washes to remove excess dye. After air‐drying, invaded and migrated cells were examined and photographed at 200× magnification. Quantitative analysis was performed by calculating the average cell count from five random fields.

### 2.7 CCK‐8 assay

Following transfection and drug treatment, GH3 cells were digested and transferred to a 96‐well plate, with 10,000 cells per well. Subsequently, fresh medium was replaced at 24, 48, and 72 h, and 10 μL of CCK‐8 solution (70‐CCK801, MultiSciences, China) was added. After incubating at 37°C for 4 h, the absorbance at 490 nm was measured using an absorbance microplate reader (HTS‐XT, Bruker Optik GmbH, Germany).

### 2.8 RNA extraction and RT‐qPCR

The SteadyPure Quick RNA Extraction Kit (AG21023, Accurate Biotechnology, China) was employed for the extraction of total RNA from human pituitary adenoma tissues and GH3 cells. Subsequently, reverse transcription was carried out using the Reverse Transcription Kit (BL696A, Biosharp, China). Subsequently, quantitative real‐time polymerase chain reaction (qRT‐PCR) was conducted using SYBR Green assays in a total reaction volume of 20 μL, employing the ABI 7500 System (Applied Biosystems). GAPDH served as the reference gene. Expression levels were quantitatively analyzed based on CT values, corrected for GAPDH expression, using the formula: 2^−∆∆CT^ [∆CT = CT (gene of interest) − CT (GAPDH), ΔΔCT = ΔCT (experimental group) − ΔCT (control group)]. All qRT‐PCR analyses were carried out in triplicate. Student's *t*‐tests were employed, and significance was determined for *p*‐values <0.05. The primer sequences are detailed in Table S2.

### 2.9 Protein extraction and western blot

Protein extraction from GH3 cells and pituitary adenoma tissues was conducted employing a total protein extraction kit (Cat. # 2140; Millipore, Billerica, MA, USA). Determination of protein concentrations was accomplished utilizing the BCA protein assay kit (23225, Pierce, Rockford, IL, USA). Subsequently, soluble proteins (20 μg) were electrophoresed on 10% sodium dodecyl sulfate polyacrylamide gels, followed by transfer to nitrocellulose membranes. Incubation with blocking buffer (5% nonfat milk) in Tris‐buffered saline/Tween‐20 (TBST) for 1 h at room temperature ensued. Membranes were then subjected to overnight probing with the respective primary antibody at 4°C, followed by three 10‐min washes with TBST. Following this, membranes were incubated with secondary antibodies conjugated to horseradish peroxidase at room temperature for 1 h, followed by three 10‐min washes with TBST. Blots were visualized through enhanced chemiluminescence, and densitometry analysis was carried out on an Amersham Imager 600 (GE). The Western blot analysis employed anti‐p38 (8690P, 1:2000; Cell Signaling Technology), anti‐phospho‐p38 (4511P, 1:2000; Cell Signaling Technology), anti‐TNFα (ab6671, 1:2000; Abcam), anti‐MMP9 (ab137867, 1:2000, Abcam), anti‐beta Actin (ab6276, 1:5000, Abcam); anti‐GAPDH (G1020V, 1:10,000; Beijing GuanXingYu Sci & Tech Co., Ltd.); anti‐ phospho‐ERK (ab184699, 1:1000; Abcam); anti‐ ERK (ab201015, 1:1000; Abcam). Grayscale scanning and semiquantitative analysis of the final data were performed using ImageJ software (https://imagej.nih.gov/ij/download.html).

### 2.10 Scanning electron microscope

The bone slices underwent a treatment in NH4OH for 30 min, followed by a 10‐min sonication process to eliminate surface cells. Subsequently, the slices underwent gradient alcohol dehydration, were air‐dried, and coated with a layer of gold powder. The examination of the bone slices was conducted using a scanning electron microscope.

### 2.11 Xenograft experiments

An in vivo assessment was conducted using a GH3 cell xenograft model to investigate the effects of oe‐TNFα transfection. Ethical approval for this study was obtained from the Ethics Committee of Beijing Tiantan Hospital. Ten male BALB/c nude mice, aged 6 weeks, were randomly allocated into two groups. Subsequently, they received subcutaneous injections on the top of their heads with 3 × 10^6^ GH3 cells, either transfected with oe‐TNFα or oe‐NC, along with serum‐free medium. After a four‐week incubation period, the mice were euthanized, and both the adenomas and mouse skulls were retrieved for subsequent experimental analyses.

### 2.12 Flow cytometric analysis

After GH3 cells were transfected with plasmids and treated with SPD304, the cells were harvested 48 h post‐transfection using 0.05% trypsin, washed with PBS. Subsequently, the cells were incubated with the Annexin V‐Alexa Flour 647/PI Kit for apoptosis analysis and the Solarbio DNA Content Quantitation Assay (cell cycle) Kit (Cat.No.CA1510) for cell cycle analysis at room temperature. Samples were analyzed using an Amnis ImageStreamX Mark II (Luminex). Data analysis was performed using FlowJo software (Becton Dickinson) and IDEAS 6.2. All experiments were repeated three times.

### 2.13 Statistical analysis

SPSS 22.0 statistical software was used for statistical evaluation. Statistical analysis of the data was performed using one way ANOVA or independent two‐sample *t* tests. *p* < 0.05 were defined as a significant difference.

## 3 RESULTS

### 3.1 Clinical and pathological characteristics of bone invasion and dural invasion

**FIGURE 1:**
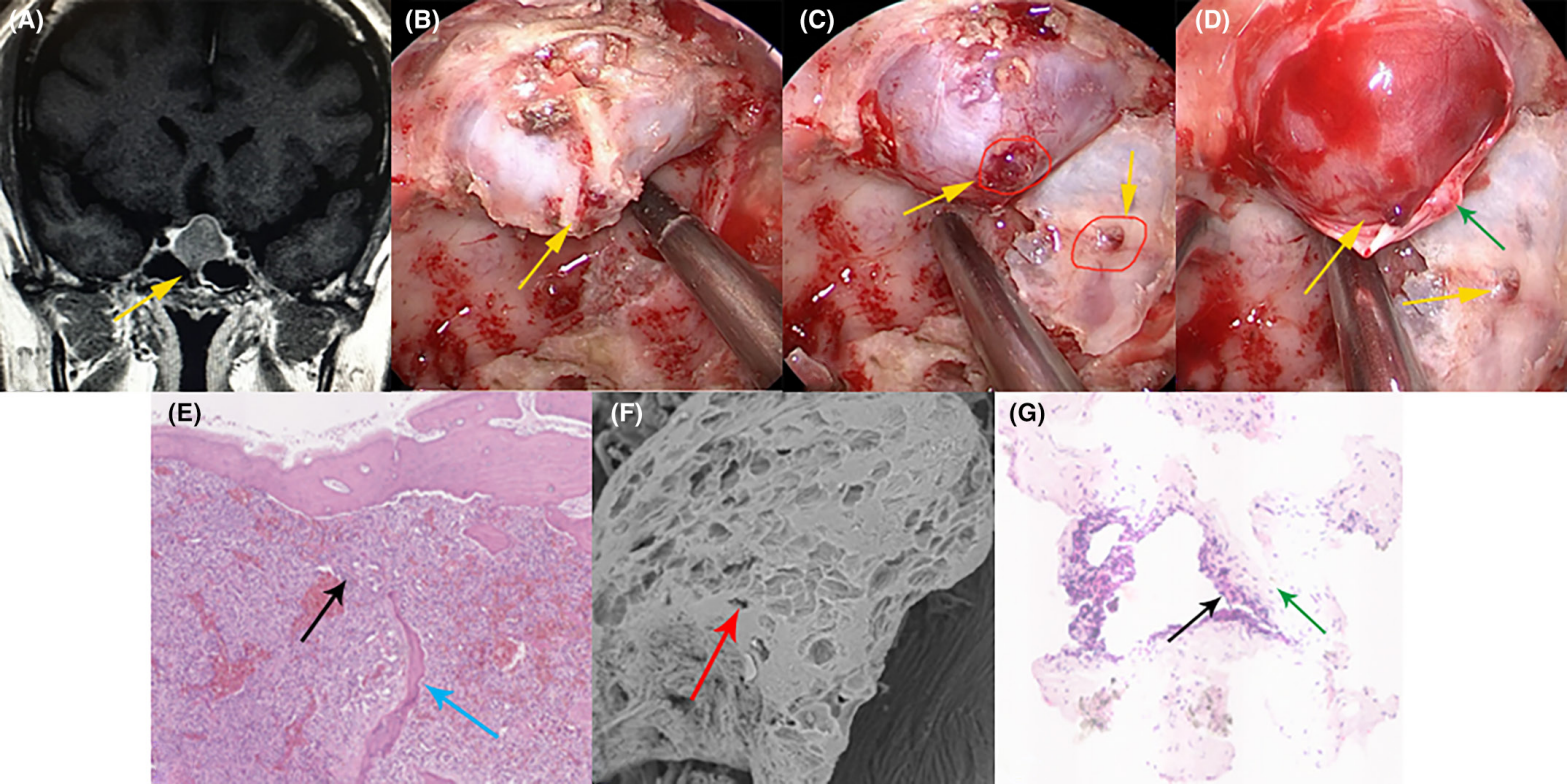
The typical imaging and pathological findings of pituitary adenomas with bone invasion. (A) Coronal MRI reveals localized destruction of the sellar base's bone. (B) During surgery, endoscopic visualization shows localized bone destruction of the sellar base. (C) The local dura mater of the sellar base is partially invaded by the tumor. (D) The pseudocapsule of the tumor is partially destroyed by the tumor invasion. (E) Bone fragments involved are subject to H&E staining (at 40x magnification), showing discontinuity in bone structure with tumor cell infiltration. (F) Under scanning electron microscopy (at 600× magnification), normal bone is observed to be disrupted into osteolytic lacunae. (G) The dura mater involved, when stained with H&E (at 40× magnification), shows discontinuity with tumor cell infiltration. The yellow arrow indicates the site of tumor invasion, the black arrow points to tumor cells, the red arrow indicates osteolytic lacunae under scanning electron microscopy, the green arrow points to the sellar floor's dura mater, and the blue arrow indicates the destroyed bone substance.

**FIGURE 2:**
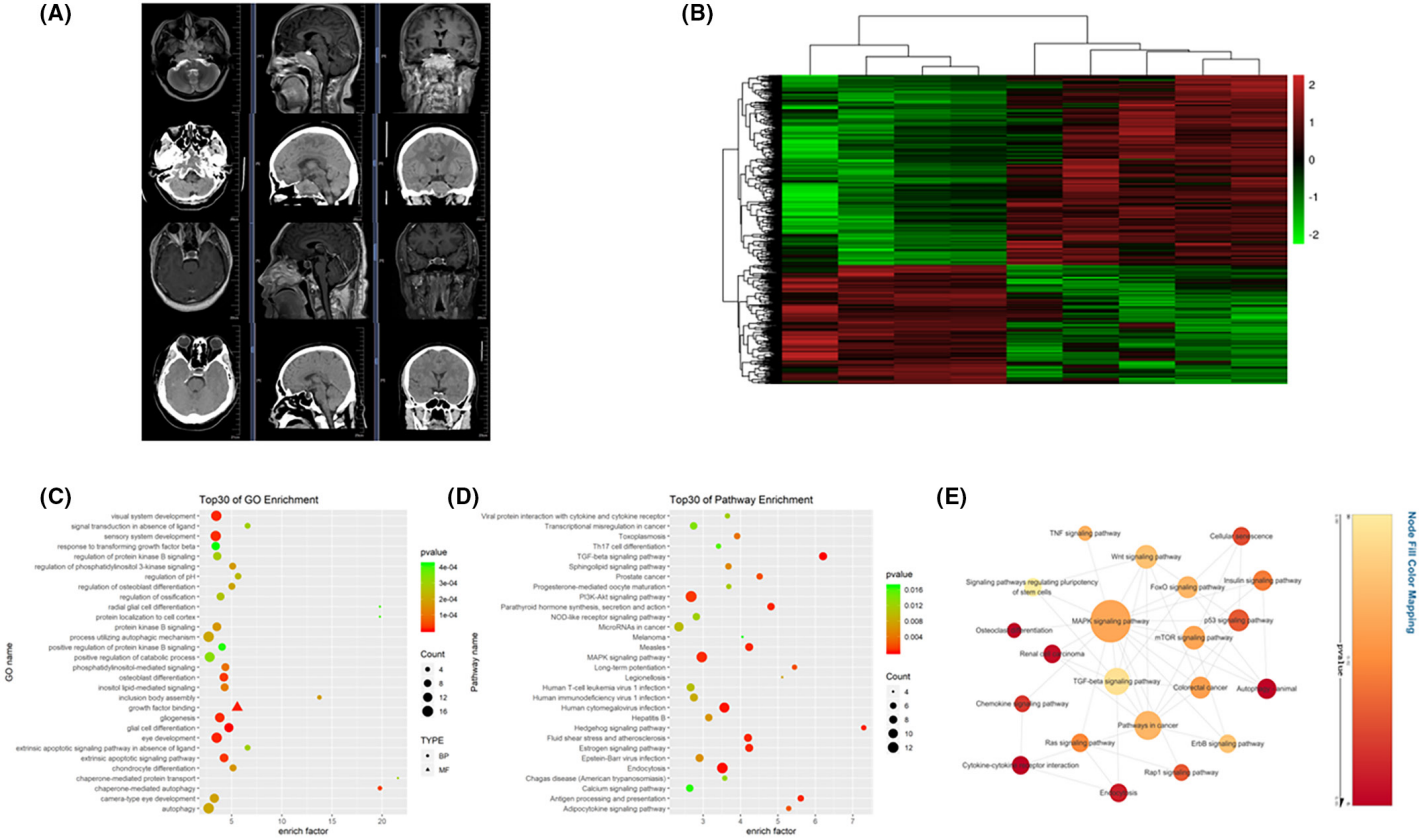
Imaging characteristics, differential gene analysis, and enrichment analysis between BIPA and NIPA. (A) The imaging performance of BIPAs and NIPAs. From the MRI and CT imaging of the BIPA, a pituitary tumor in the sella region is visible (first and second rows), with the tumor encircling the internal carotid artery. The tumor has invaded the surrounding bone tissue, causing discontinuity in the bone cortex and forming small bone fragments. From the MRI and CT imaging of the NIPA, a pituitary tumor in the sella turcica region is visible (third and fourth rows), but the tumor does not involve the internal carotid artery or the surrounding bone tissue. (B) Heat map showing the expression profiles of mRNAs between the two groups. Differentially expressed mRNAs (fold change >2 or <0.5 and *p* < 0.05) between the two groups were analyzed using hierarchical clustering. (C) GO annotation of differentially expressed mRNAs with the top 30 significant enrichments covering domains of biological processes, cellular components, and molecular functions. (D) KEGG pathway analysis of mRNAs enriched in the top 30 pathways according to the *p* value. (E) Cytoscape pathway act network: pathway act network according to the overlap of common differentially expressed molecules in the top 30 significant canonical pathways. The size of the circle represents the degree of association.

From preoperative MRI and CT scans (Figures 1A and 2A), we observed that the characteristic features of BIPA included destruction of the base of the sella turcica. Intraoperatively, direct visualization revealed bone destruction (Figure 1B,C), and the integrity of the dura mater at the base of the sella turcica was disrupted by the tumor. Electron microscopy also showed bone absorption (Figure 1F). HE staining of the dura mater specimens showed that tumor cells invaded the dura mater, leading to disruption of its structural integrity (Figure 1E,G).

### 3.2 Differential expressed gene analyze and enrichment analyze

Compared with the NIPAs, 1118 mRNAs were significantly differentially expressed, including 703 upregulated and 415 downregulated mRNAs. Hierarchical clustering showed that the expression patterns of the mRNA between two groups were obviously distinguishable (Figure 2B). GO analysis was performed to study the biological processes, cellular components, and specific molecular functions of all differentially expressed mRNAs. We performed GO analysis of the mRNAs that were differentially expressed between the two groups. The results showed that the biological processes were mainly about eye development and visual system development. KEGG pathway enrichment analysis revealed that differential genes between BIPA and NIPA were significantly enriched in the osteoclast differentiation pathway and TNF signaling pathway and MAPK signaling pathway (Figure 2C,D). Pathway interaction analysis further identified the MAPK signaling pathway in a pivotal position among these enriched pathways (Figure 2E).

### 3.3 TNFα and proteins in the MAPK pathway are upregulated in BIPA

**FIGURE 3:**
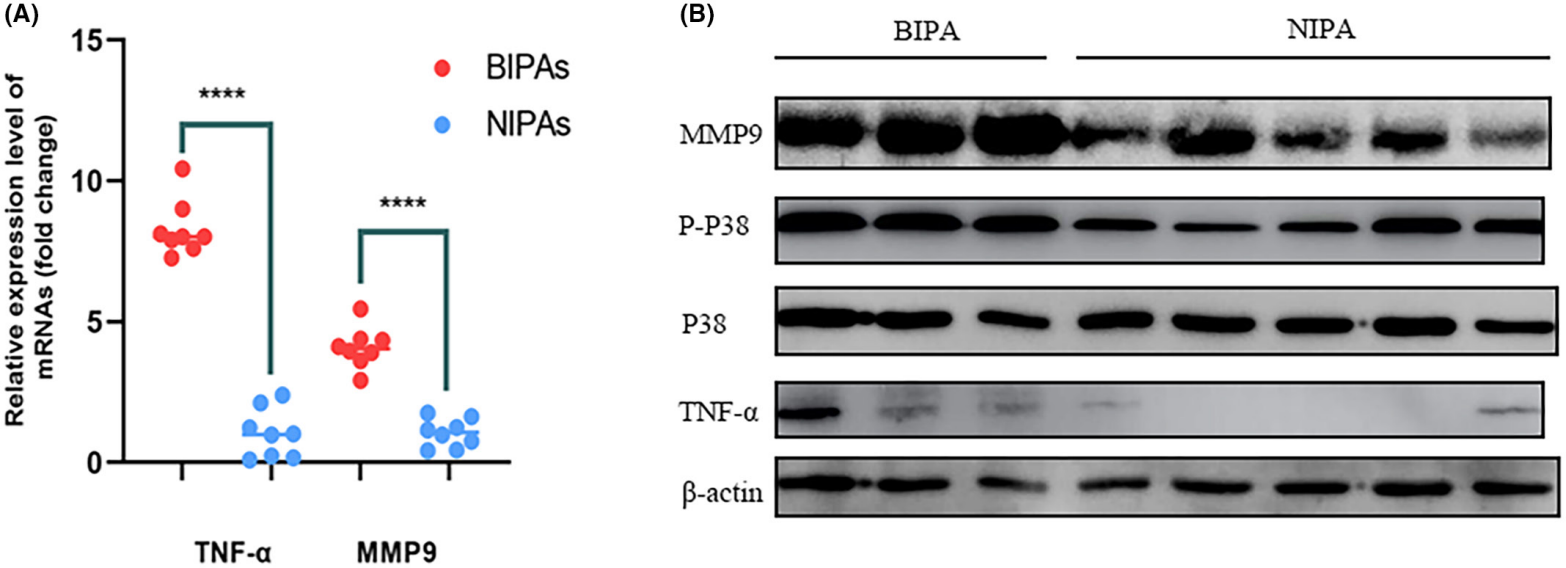
Expression levels of TNFα and MMP9 between BIPA and NIPA. RT‐qPCR (A) and Western blot (B) were used to detect the expression of TNF‐α, MMP9 and p‐p38 in BIPAs and NIPAs. ****p < 0.0001.

RT‐qPCR analysis of tumor specimens revealed elevated mRNA expression levels of TNFα and MMP9 in BIPA (Figure 3A). Western blot analysis indicated increased protein expression levels of p‐p38, MMP9, and TNFα (Figure 3B) in BIPA.

### 3.4 Overexpression of TNFα upregulates the expression of proteins in the MAPK pathway, promoting the proliferation migration and invasion of GH3 cells

**FIGURE 4:**
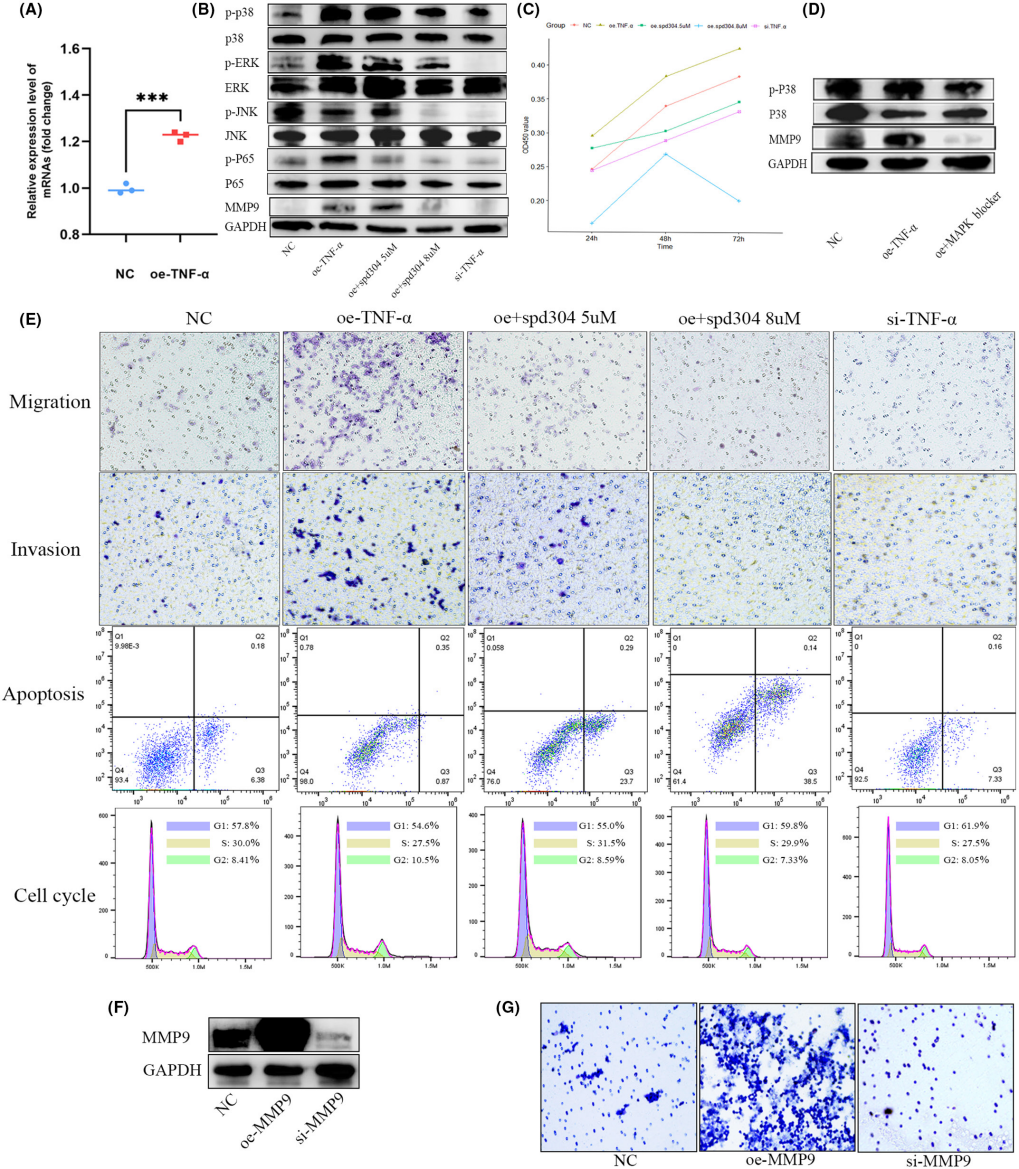
The effect of TNF‐α in promoting membrane invasion was verified in GH3 cell lines. (A, B) RT‐qPCR and Western blot analyses confirmed the efficacy of the overexpression and interference plasmids for TNF‐α, ***p < 0.001. (C) CCK8 assay demonstrated that TNF‐α overexpression promoted the proliferation of GH3 cells, which was inhibited by spd304. (D) The P38 MAPK inhibitor significantly inhibited the expression of p‐P38 and MMP9. (E) Overexpression of TNF‐α enhanced the proliferation migration and invasion of GH3 cells, effects that were mitigated by spd304. (F) Western blot verified the effects of MMP9 overexpression or knockdown. (G) High expression of MMP9 significantly promoted the invasion of GH3 cells.

Based on different plasmids, GH3 cells were divided into a negative plasmid control group (NC), a TNFα overexpression group (oe‐TNF‐α), TNF overexpression + SPD304 5 μM group (oe + SPD304 5 μM), TNF overexpression + SPD304 8 μM group (oe + SPD304 8 μM), and a TNF α RNAi group (si‐TNF‐α). Forty‐eight hours after transfection, RT‐qPCR and Western blot analyses were conducted, revealing that TNFα overexpression in GH3 cells upregulates MMP9 and key molecules in the MAPK and NF‐KB pathways, including p‐p38, p‐Erk, p‐JNK, and p‐P65. Conversely, siRNA downregulates the expression of these molecules at both the mRNA and protein levels. The P38 MAPK inhibitor significantly inhibited the expression of p‐P38 and MMP9 in GH3 cells (Figure 4D). Additionally, the selective inhibitor of TNFα, SPD304, suppresses the upregulation of MAPK pathway proteins induced by TNFα (Figure 4A,B). Cell proliferation, migration, and invasion experiments demonstrated that TNFα overexpression promotes proliferation, migration, and invasion of GH3 cells, while SPD304 inhibits this effect induced by TNFα (Figure 4C,E).

### 3.5 Overexpression of MMP9 promoted invasion of GH3 cells

We used plasmids and small interfering RNA (siRNA) to upregulate or knock down MMP9 protein expression and validated their efficacy through western blot analysis (Figure 4F). Subsequently, Transwell plates containing matrix gel were used to validate the invasive ability of GH3 cells in each group. The results indicated that overexpression of MMP9 significantly promoted the invasiveness of GH3 cells (Figure 4G).

### 3.6 Flow cytometry analysis

Flow cytometry shows that overexpression of TNF‐α can significantly increase the proportion of GH3 cells in the G2 phase and inhibit apoptosis, while interfering with the expression of TNF‐α can have the opposite effect. SPD304 can significantly inhibit the effects of overexpressing TNF‐α, promoting apoptosis and suppressing the proportion of cells in the G2 phase (Figure 4E).

### 3.7 In vivo experiments confirm that overexpression of TNF‐α can promote tumor proliferation and bone invasion

**FIGURE 5:**
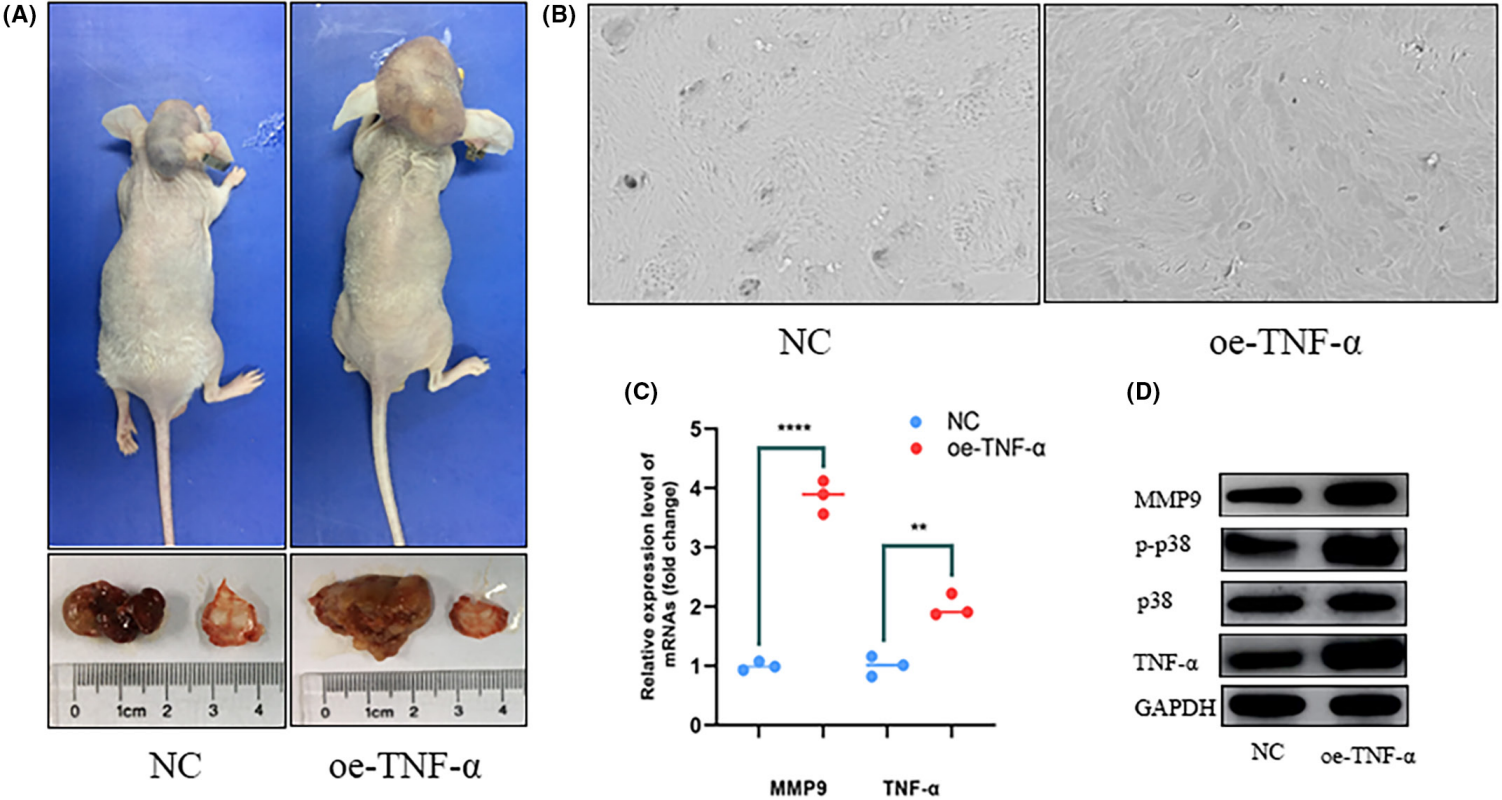
Overexpression of TNF‐α promoted tumorigenesis and tumor progression in vivo. (A) Mice models, tumor volume and bone slices. (B) Scanning electron microscope results of bone slices (1000×). (C) RT‐qPCR analyses measured relative MMP9andTNF‐α expression levels, ***p < 0.001, **p < 0.01. (D) Western blot was used to detect the expression of TNF‐α and MMP9 in mice models.

The GH3 cells, transfected with TNFα and its negative control plasmid, were injected subcutaneously into the scalp of mice. Four weeks later, the mice were euthanized. It was observed that the group with TNFα overexpression had significantly larger tumor volumes (Figure 5A). Electron microscopy scans indicated an increased number of bone resorption nests in the TNFα overexpression group (Figure 5B). PCR analysis demonstrated elevated mRNA expression levels of TNFα and MMP9 in the TNFα overexpression group (Figure 5C). Western blot analysis of tumor specimens revealed higher protein expression levels of TNFα, phosphorylated p38 (p‐p38), and MMP9 in the TNFα overexpression group (Figure 5D).

## 4 DISCUSSION

Invasive pituitary adenomas represent a challenging subset of pituitary adenomas due to their intricate treatment landscape. Characterized by rapid tumor growth, drug resistance, and high recurrence rates, these tumors pose significant therapeutic challenges. Prior studies on invasive pituitary adenomas had predominantly focused on cavernous sinus invasion, leaving more resistant tumors like bone‐invasive pituitary adenomas (BIPAs) underexplored. Intraoperative visualization and HE staining revealed that prior to invading bone, adenoma cells needed to breach the dura mater. The specific mechanisms underlying adenoma cell invasion of the dura mater remain unclear.

To elucidate the specific mechanisms of membranous invasion, we conducted transcriptome microarrays on BIPA and NIPA samples. Subsequent enrichment analysis of differentially expressed genes revealed a predominant enrichment in pathways related to osteoclast differentiation, the TNF signaling pathway, and the MAPK signaling pathway. Following this, we conducted PCR and western blot validation on tumor specimens from both BIPA and NIPA. Our findings revealed a significant upregulation of TNFα and MMP9 at both the mRNA and protein levels of TNFα and the MAPK pathway. These findings corroborate our microarray results, suggesting that the TNFα and MAPK pathways may play a crucial role in the process of membranous invasion in pituitary adenomas.

TNF‐α is a crucial inflammatory factor with diverse biological functions, including the regulation of inflammatory responses, immune reactions, cell apoptosis, and antiviral responses.^7^ TNFα has been confirmed to promote the growth, migration, and invasion of various tumors, such as breast cancer, colorectal cancer, and gastric cancer.^8^, ^9^, ^10^ Similarly, in pituitary adenomas, TNF‐α has been confirmed to be associated with tumor invasion, exhibiting elevated expression in invasive pituitary adenomas.^5^, ^11^ It was reported that TNF‐α receptors are widely distributed in human cells, and upon binding with TNFα, they can activate the MAPK signaling pathway.^12^, ^13^, ^14^

The MAPK family is comprised of three subfamilies: extracellular signal‐regulated kinases (ERKs), c‐Jun N‐terminal kinases (JNKs), and p38 MAPKs.^15^ The MAPK signaling pathway is widely distributed in various organs, tissues, and cells, playing a crucial role in cell proliferation, the cell cycle, apoptosis, migration, and invasion processes.^16^, ^17^, ^18^, ^19^ TNFα can activate members of the MAPK family, including JNK, ERK, and p38. The MAPK signaling pathway is essential in the occurrence and development of pituitary adenomas, where miR‐16 can inhibit the ERK/MAPK signaling pathway to suppress the proliferation of pituitary adenoma cells.^20^ Additionally, studies have reported that TNF‐α can induce MMP9 expression through the MAPK pathway, and MMP9 is a crucial molecule in degrading the extracellular matrix and invading membranous structures.^21^, ^22^, ^23^, ^24^ Our results indicated that MMP9 significantly promoted the invasion of GH3 cells. MMP‐9 is the first matrix metalloproteinase found to be significantly upregulated in pituitary adenomas infiltrating the cavernous sinus,^25^ and subsequent studies have further confirmed its crucial role in the invasion of pituitary adenomas.^26^, ^27^

SPD304 is a small‐molecule inhibitor of TNFα that facilitates the subunit dissociation of TNFα trimers. This compound exhibits inhibitory activity against TNF‐α in both biochemical and cell‐based assays, with median inhibitory concentrations of 22 and 4.6 μM, respectively.^28^ The efficacy of SPD304 has been validated in multiple studies.^29^, ^30^, ^31^

We further validated the effects of TNFα in the GH3 cell line and observed that TNFα overexpression upregulates the expression of proteins such as MMP9, p‐p38, and p‐ERK. The P38 MAPK inhibitor significantly inhibited the expression of p‐P38 and MMP9. The inhibitor SPD304 significantly inhibits this effect of TNFα in cell proliferation, migration, and invasion assays on GH3 cells. Finally, in vivo experiments involving subcutaneous injection of GH3 cells into the scalp revealed that TNFα overexpression in GH3 cells leads to significantly increased tumor volume and more pronounced bone destruction. Western blot experiments on adenoma specimens confirmed the upregulation of MAPK pathway proteins and MMP9. These results validate our previous hypothesis that TNF‐α secreted by BIPA can act on itself in an autocrine manner, activating the MAPK/MMP9 pathway, enhancing its own invasiveness, and leading to membrane invasion and bone invasion.

**FIGURE 6:**
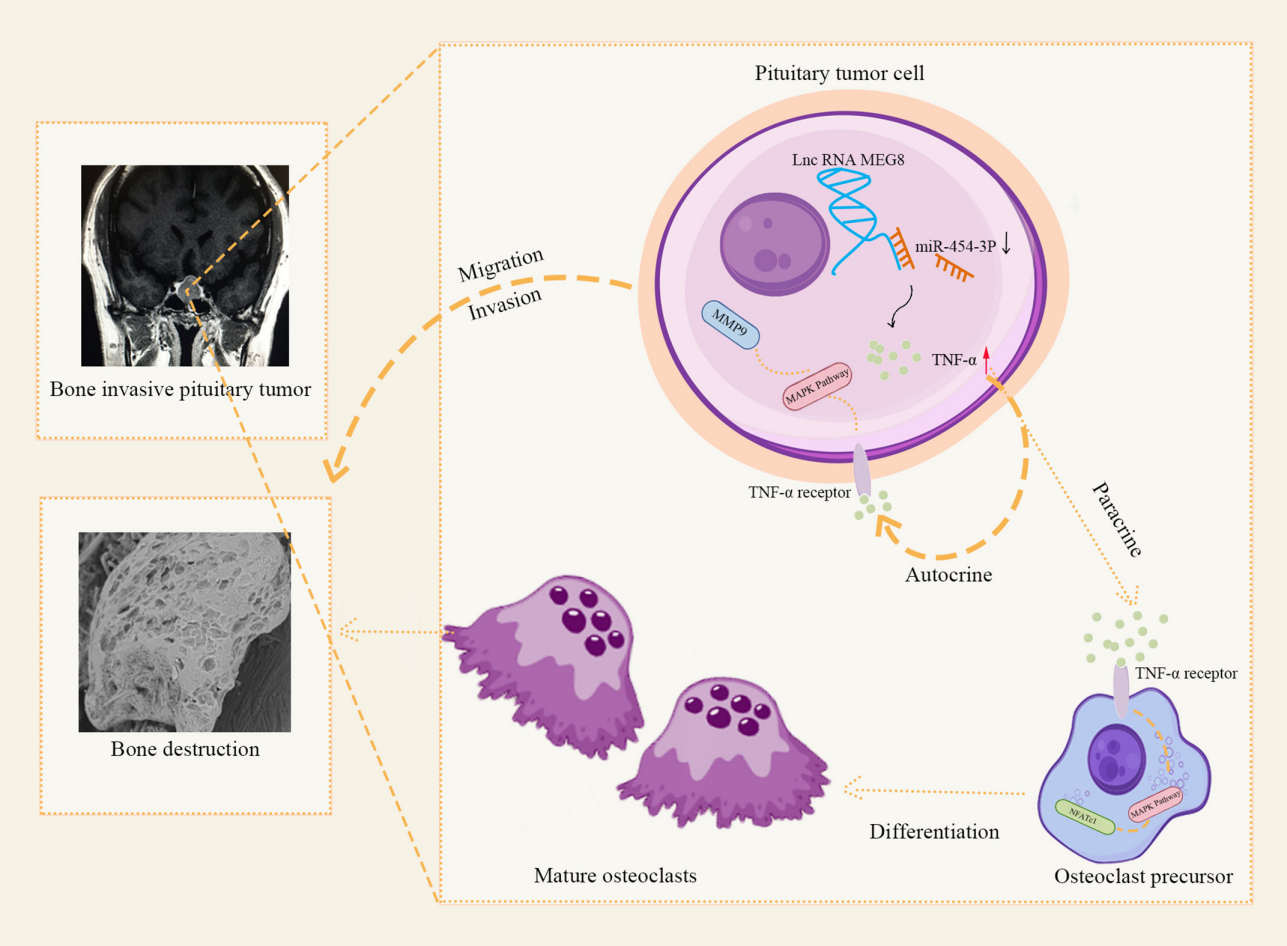
The two approaches of TNF‐α in promoting bone invasion: autocrine and paracrine.

In our previous studies, we discovered that tumor cells in BIPA promote osteoclast differentiation and subsequently induce bone destruction by secreting excessive TNFα.^5^, ^6^ In this study, we further confirmed that the secretion of TNFα from BIPA not only promotes bone invasion by facilitating the differentiation of osteoclasts but also acts on pituitary adenoma cells in an autocrine manner, enhancing their proliferation, invasion, and migration capabilities. This results in the breach of the dura mater, accelerating bone invasion. These two approaches to promoting bone invasion are summarized in Figure 6.

## 5 CONCLUSION

BIPA exhibits higher levels of TNFα secretion. The excessive TNFα acts on pituitary adenoma cells in an autocrine manner, activating the MAPK signaling pathway and increasing the expression of MMP9. This enhances the invasive capability of the pituitary adenoma cells, accelerates the process of membrane invasion, and ultimately leads to increased bone invasion in BIPA. SPD304, as a small‐molecule inhibitor of TNFα, can inhibit the upregulation of TNFα on MAPK and MMP9, thereby reducing the membrane invasion capability and bone invasion ability of pituitary adenoma cells. It holds promise as a novel therapeutic strategy for BIPA.

## 6 LIMITATIONS

This study also has its limitations; first, GH3 cells were used as the research subject instead of primary pituitary adenoma cells. This might have impacted the reliability of the results, but currently, we are unable to perform functional experiments using primary pituitary adenoma cells. We are continuously trying to culture primary cells and exploring suitable conditions, hoping to achieve a breakthrough in this technology in the future. Second, the efficacy of SPD304 needs further validation to aid in clinical treatment.

## AUTHOR CONTRIBUTIONS

**Xinzhi Wu:** Conceptualization, Methodology, Validation, and Writing. **Lei Gong:** Formal analysis, Visualization, and Validation. **Jiwei Bai:** Methodology and Data curation. **Bin Li:** Methodology and Validation. **Chuzhong Li:** Methodology. **Yazhuo Zhang:** Conceptualization, Methodology, and Supervision. **Haibo Zhu:** Writing—review and editing, Project administration, and Funding acquisition.

## FUNDING INFORMATION

This Study funded by the National Natural Science Foundation of China (grant number 82103028).

## CONFLICT OF INTEREST STATEMENT

The authors have no conflicts of interest to declare.

## Supporting information


Table S1.–S2.

## Data Availability

The data that support the findings of this study are available from the corresponding author upon reasonable request.
