# Supplementary material for: TNF‐α can promote membrane invasion by activating the MAPK/MMP9 signaling pathway through autocrine in bone‐invasive pituitary adenoma
Source: CNS Neurosci Ther. 2024 May 13;30(5):e14749. doi: 10.1111/cns.14749 (PMC11090077; doi:10.1111/cns.14749)
Supplement: Supplementary file 1 — Table S1.–S2. [file CNS-30-e14749-s001.docx]

**Table S1 Clinical information of transcriptive microarrays patients**

| Type | Sex | Age  (years) | Pathological type | Knosp classification (Left, Right) | | Hardy  Classification | |
| --- | --- | --- | --- | --- | --- | --- | --- |
| BIPA | M | 47 | NFPA | 2 | 2 | 4 | B |
| BIPA | M | 56 | NFPA | 1 | 2 | 4 | A |
| BIPA | F | 56 | NFPA | 2 | 1 | 4 | 0 |
| BIPA | M | 42 | NFPA | 2 | 2 | 4 | A |
| BIPA | F | 44 | NFPA | 2 | 2 | 4 | 0 |
| NIPA | M | 63 | NFPA | 1 | 1 | 2 | A |
| NIPA | F | 57 | NFPA | 1 | 1 | 2 | 0 |
| NIPA | M | 47 | NFPA | 1 | 1 | 2 | A |
| NIPA | F | 34 | NFPA | 0 | 1 | 2 | A |

**Table S2 PCR primers of mRNAs used for qRT-PCR**

| Gene symbol | Gene type | Forward primer | Reverse primer |
| --- | --- | --- | --- |
| hTNF | mRNA | 5‘AAACAATGCTGATTTGGTGAC3‘ | 5‘GCAAACTTTATTTCTCGCCACT3‘ |
| hMMP9 | mRNA | 5’GCCACTACTGTGCCTTTGAGTC3’ | 5’ CCCTCAGAGAATCGCCAGTACT3’ |
| hGAPDH | mRNA | 5‘TGACTTCAACAGCGACACA3’ | 5‘CACCCTGTTGCTGTAGCCAAA3’ |
| rTNF | mRNA | 5‘CCAACTCCGGGCTCAGAATT3‘ | 5‘TCCAGTGAGTTCCGAAAGCC3‘ |
| rMMP9 | mRNA | 5’TCTGCCTGCACCACTAAAGG3’ | 5’TCGGCTCGAGTAGGACAGAA3’ |
| rGAPDH | mRNA | 5‘GCTGGTGCTGAGTATGTCGT3’ | 5‘TCACAAACATGGGGGCATCA3’ |
